# Antibiotic resistance profiles of seven genomospecies of *Corynebacterium jeikeium* analyzed by whole genome sequencing

**DOI:** 10.1128/jcm.00418-25

**Published:** 2025-09-03

**Authors:** Michaela Fischer-Wellenborn, Frank Imkamp, Valéria Pereira Pires, Reinhard Zbinden, Nicolas Personnic

**Affiliations:** 1 Institute of Medical Microbiology, University of Zurich 27217, Zürich, Switzerland; Universitat Munster, Münster, Germany

**Keywords:** *Corynebacterium jeikeium*, whole genome sequencing, MALDI-TOF mass spectrometry, genomospecies, phylogeny, antimicrobial susceptibility testing, multi-drug resistance

## Abstract

*Corynebacterium jeikeium* is an opportunistic human pathogen with high genomic diversity. In the past, multi-drug resistance has been considered a hallmark of *C. jeikeium*. However, some strains of *C. jeikeium* are fully susceptible and thus, their reclassification would avoid the empirical therapy with glycopeptides. One hundred eighty-six clinical isolates and four reference isolates identified as *C. jeikeium* between 1994–1999 and 2012–2019, respectively, were analyzed by matrix-assisted laser desorption/ionization time of flight mass spectrometry (MALDI-TOF MS) and 16S ribosomal RNA (16S rRNA) gene sequence analysis. A total of 153 confirmed *C. jeikeium* isolates was further analyzed by whole genome sequencing (WGS) and by disk diffusion antimicrobial susceptibility testing (AST). WGS and subsequent core genome single nucleotide polymorphism-based phylogenetic analysis segregated the isolates into seven phylogenetic clusters. The average nucleotide identity between the clusters was <95%, qualifying them as separate *C. jeikeium* genomospecies. Overall, AST profiles of all 153 isolates were heterogeneous for six of the 16 antibiotics tested. Notably, genomospecies 6 and 7 displayed mainly comparably small inhibition zones. For the other 10 antibiotics, all genomospecies displayed either small or large inhibition zones, respectively. Our study of *C. jeikeium* clinical isolates revealed a relatively complex phylogenetic structure. As soon as clear phenotypic traits can be attributed to the seven genomospecies by routine diagnostic tests, genomospecies encompassing mainly antibiotic-susceptible strains should be reclassified to fine-tune potential treatment strategies.

**IMPORTANCE:**

*Corynebacterium jeikeium* isolates typically display multi-drug resistance (MDR), frequently triggering empirical therapies with glycopeptides. However, some strains are susceptible to a broad range of antibiotics. In our study, analysis of a large collection of *C. jeikeium* by whole genome sequencing (WGS) and antibiotic resistance testing revealed genomic diversity, and some correlations between MDR phenotypes and specific genomospecies. While WGS is not yet a routine method, identification of MDR genomospecies may confirm the need for glycopeptide therapy. Identification of a less-resistant genomospecies would have even a higher clinical impact, as unnecessary therapies with glycopeptides are avoided, thereby reducing the selection for vancomycin-resistant enterococci. In the future, databases of diagnostic tools, e.g., MALDI-TOF, may be expanded to allow for the differentiation of genomospecies. Furthermore, rational taxonomic reclassification of some *C. jeikeium* genomospecies would help clinicians to fine-tune potential treatment strategies.

## INTRODUCTION

While corynebacteria are commonly viewed as non-pathogenic, their association with increased antibiotic resistance levels may contribute to disease development. Until 1990, corynebacteria were not subject to in-depth differentiation in our routine microbiological laboratory. *Corynebacterium diphtheriae* was identified based on morphological (polar bodies in methylene Neisser strain) and biochemical characteristics (growth on cystine tellurite blood agar as black colonies due to the reduction of tellurite to tellurium). Other Gram-positive rods were reported as coryneform bacteria except those with resistances to many antibiotics. In this context, it was common practice to categorize corynebacteria with multiple antibiotic resistances as *Corynebacterium jeikeium* (1). Indeed, *C. jeikeium* is resistant to a broad spectrum of antibiotics, including aminoglycosides, β-lactams, macrolides, quinolones, rifampicin, and tetracycline (2–6). In the 1990s, an identification algorithm derived from the Hollis-Weaver scheme was introduced in our routine laboratory for aerobic, fast-growing Gram-positive rods (7). The combination of biochemical tests and the analysis of cellular fatty acids allowed the reclassification of various poorly defined corynebacteria (3, 8). Furthermore, a commercial miniaturized system (API Coryne, bioMérieux, Marcy l'Etoile, France) with conventional biochemical methods allowed a more rapid identification of corynebacteria (9). Importantly, the identification of all corynebacteria to species level revealed antibiotic-susceptible *C. jeikeium* strains but also multi-resistant strains of the so-called group G2 (1), which was later renamed *Corynebacterium tuberculostearicum* (10). *C. jeikeium* is lipophilic but distinct from the other lipophilic species, i.e., *C. tuberculostearicum* (10), *Corynebacterium macginleyi* (11), *Corynebacterium accolens* (12), and the two lipophilic urease-positive genomospecies described by Riegel et al. (11). Therefore, the correct identification of lipophilic coryneform Gram-positive rods as *C. jeikeium* was based on specific biochemical properties as well as antimicrobial susceptibility (13).

*C. jeikeium* is a well-known opportunistic pathogen causing wound infections, sepsis, and other infections, particularly in immunocompromised, hospitalized patients (1). In addition, various life-threatening *C. jeikeium*-related nosocomial infections, such as endocarditis, septicemia, pneumonia, peritonitis, osteomyelitis, meningitis, infections of hemodialysis catheters, and soft tissue infection have been described (2–4, 14–16). Lipids produced by human skin sustain lipophilic *C. jeikeium* as part of the skin flora (6, 8, 17). In a 10-year survey, *C. jeikeium* was the most frequently isolated *Corynebacterium* sp. from blood culture, half of which were indeed associated with bloodstream infections (18). With the exception of *Corynebacterium amycolatum*, other *Corynebacterium* spp. isolated from blood were predominantly skin contaminants (18). During the same period, *C. jeikeium* could not be isolated from patients with suspected orthopedic infections (18); other reports support the observation that *C. jeikeium* is less common in bone and joint infections than *C. amycolatum* and *C. striatum* (19, 20). In immunocompromised patients, as well as in patients under invasive medical care, outcomes of *C. jeikeium* infections are often poor, with a reported overall mortality rate of 33% in case of endocarditis, even after treatment with appropriate antibiotics (15).

A comprehensive understanding of *C. jeikeium* virulence as well as of its antibiotic resistance mechanisms was hampered by the preference given to clinical case studies at the expense of experimental microbiology. In addition, the difficulties in classifying isolates hindered any further comprehensive studies. Based on standard analytical methods, *C. jeikeium* isolates generally share highly similar biochemical patterns. However, early works based on DNA-DNA hybridization indicated an inherent genomic diversity among *C. jeikeium* isolates and, importantly, heterogeneity regarding bacterial antibiotic resistance (13, 21, 22). Whole genome sequencing (WGS) of a small collection of *C. jeikeium* clinical isolates revealed a high degree of divergence among the strains, which could be divided into four genetically distinct *C. jeikeium* subgroups; however, these phylogenetic clusters displayed similar metabolic or antibiotic susceptibility profiles (23).

Here, we aimed to untangle the phylogeny and resistance profiles of a large collection of *C. jeikeium* clinical isolates, collected between 1994–2019, using WGS and comprehensive antibiotic susceptibility testing. A practical aspect of our study was to correlate resistance patterns with different genomospecies to reclassify susceptible strains that do not need glycopeptides, e.g., vancomycin or teicoplanin. We expect our work to fine-tune treatment strategy and to serve as a platform for future analysis of resistance mechanisms occurring in different phylogenetic clusters of *C. jeikeium*.

## MATERIALS AND METHODS

### Isolates and growth conditions

The entity of clinical isolates of corynebacteria analyzed in this study (*n* = 186) is listed in the Table S1. The strains were isolated at the Institute of Medical Microbiology (IMM, University of Zurich, Switzerland) between 1994 to 1999 (*n* = 141) and 2012 to 2019, i.e., Cj 146 to Cj 190 (*n* = 45), respectively, from patients hospitalized in the Zurich area. The first identification of the isolates of 1994–1999 was based on conventional methods (7). Isolates collected between 2012–2019 were initially identified by conventional methods and subsequent confirmation by MALDI-TOF and 16S rRNA gene. Three *C. jeikeium* clinical isolates belonging to distinct DNA groups were kindly provided by P. Riegel (Institute of Bacteriology, University Louis Pasteur, Strasbourg, France) (Cj-74, i.e., B30049-DNA group B; Cj-72, i.e., B15507-DNA group C and Cj-75, i.e., B6225-DNA group D) (13); the *C. jeikeium* type strain (Cj-56, ATCC 43734) was purchased at the American Type Culture Collection (ATCC, Manassas, Virginia, United States). The isolates were stored at −80°C and routinely cultivated on 5% sheep blood agar plates (bioMérieux), followed by subsequent cultivation on sheep blood agar plates supplemented with 0.5% Tween (Hänseler AG, Herisau, Switzerland). Plates were incubated for 20 to 24 h at 37°C with 5% CO_2_.

### Matrix-assisted laser desorption/ionization time of flight mass spectrometry (MALDI-TOF MS)

Single bacterial colonies were transferred onto a polished steel MSP 96 target (Bruker Daltonics GmbH, Bremen, Germany) using a toothpick. Prior to the addition of 1 µL saturated α-cyano-4-hydroxycinnamic acid (HCCA; Bruker) as matrix solution, cells were lysed by overlaying them with 1 µL 70% formic acid. MALDI-TOF MS identification of strains was performed using the MBT smart MALDI-TOF MS BioTyper and the BioTyper software version 4.1 (Bruker).

### Whole genome sequencing (WGS)

DNA extraction from freshly grown cells was performed using the DNeasy UltraClean Microbial Kit (250) (QIAGEN GmbH, Hilden, Germany) according to the manufacturer’s instructions. For library preparation, 30 ng of DNA from each isolate was processed using the QIAseq FX DNA Library kit (QIAGEN GmbH, Hilden, Germany) following the producer’s recommendations. Finally, sequencing library quality and size distribution were analyzed on a fragment analyzer automated capillary electrophoresis (CE) system (Advanced Analytical Technologies Inc., Heidelberg, Germany), according to the manufacturer’s instructions using the fragment analyzer 474 high sensitivity (HS) next generation sequencing (NGS) kit. Sequencing libraries were pooled in equimolar concentrations and paired-end sequenced (2 × 150 bp) on an Illumina MiSeq platform (Illumina, San Diego, CA, USA).

### Bioinformatic analyses

Raw sequencing reads (FASTQ) were trimmed and quality filtered using Trimmomatic (24). The isolate’s 16S rRNA sequences were extracted from sequencing data using ARIBA (25). Molecular identification of isolates was achieved by using BLAST (26) and querying the NCBI database with the 16S rRNA gene sequences, respectively. SPAdes (27) was used for *de novo* assembly of sequencing data. Resulting CONTIGS served as basis for gene prediction and annotation as well as genome size approximation using PROKKA (28); *C. jeikeium* K411 served as reference (6). For comparison, the sequences of 13 isolates (Cj-14566, Cj-16348, Cj-19409, Cj-21382, Cj-30184, Cj-30952, Cj-37130, Cj-38002, Cj-47444, Cj-47445, Cj-47446, Cj-47447, and Cj-47453) published by Salipante et al. were integrated to the phylogenetic analyses (23). Core genome single-nucleotide polymorphisms (SNPs) calling was performed using Snippy (https://github.com/tseemann/snippy). A core genome SNPs-based neighbor-joining phylogenetic tree was generated by SEAview (https://doua.prabi.fr/software/seaview3) using 1,000 bootstrap replications and visualized using Figtree (http://tree.bio.ed.ac.uk/software/figtree/). Average nucleotide identities (ANIs) were determined using the FastANI algorithm (29). Isolates were considered to belong to the same (genomo-)species when sharing an ANI value of ≥95% (23, 30, 31).

### Antimicrobial susceptibility testing (AST)

AST was performed using the Kirby-Bauer disk diffusion method according to EUCAST guidelines with Mueller-Hinton agar +5% defibrinated horse blood and 20 mg/L β-NAD (MH-F) (BioMérieux). Plates were incubated for 18 ± 2 h (35°C, 5.5% CO_2_). Isolates with insufficient growth after 16–20 h incubation were reincubated, and inhibition zones were read after a total of 40–44 h incubation (32). Inhibition zones of the disks of penicillin (PEN, one unit), ceftriaxone (CRO, 30 µg), clindamycin (CLI 2 µg), erythromycin (ERY, 15 µg), trimethoprim-sulfamethoxazole (SXT, 25 µg), linezolid (LZD, 10 µg), tetracycline (TET, 30 µg), tigecycline (TGC, 15 µg), vancomycin (VAN, 5 µg), teicoplanin (TEC, 30 µg), meropenem (MEM, 10 µg), imipenem (IPM, 10 µg), ciprofloxacin (CIP, 5 µg), gentamicin (GEN, 10 µg), rifampicin (RIF, 5 µg), and fusidic acid (FA, 10 µg) (i2a, Axon Lab AG, Baden, Switzerland) were recorded and electronically archived using the Sirweb/SirScan 2000 Automatic system (i2a, Montpellier, France). Isolates were categorized as susceptible (S) or resistant (R) to an antibiotic according to the version 15 of EUCAST clinical breakpoint (CBP) tables provided for *Corynebacterium* spp. other than *Corynebacterium diphtheriae* and *Corynebacterium ulceran*s, i.e., 20 mm for clindamycin, 25 mm for linezolid, 24 mm for tetracycline, 17 mm for vancomycin, and 30 mm for rifampicin; for erythromycin the CBP for *C. diphtheriae* (24 mm) was applied (32). For penicillin and ciprofloxacin, isolates with a CBP of 12 and 25 mm, respectively, were classified as resistant (R) or susceptible at increased doses (I) (32). For this study, we calculate the category I as S for susceptible. Since no CBPs are available for the other antibiotics, we used the distribution of inhibition zones, respectively, to visually separate the group of bacteria with larger inhibition zones from the group with small inhibition zones, which approximates an epidemiological threshold (eyeball ECOFF) (Kahlmeter G, EUCAST workshop-ECCMID 2013). Isolates of a species that exhibit inhibition zones at or above the ECOFF are considered to be wild type (WT), while those with inhibition zones below the ECOFF constitute the non-wild-type population (non-WT) (33). For our investigation, we consider the WT population to be susceptible and the non-WT population to be resistant. The isolate’s distribution for each tested antibiotic was defined as unimodal when at least 80% of the isolates were either resistant or susceptible.

### Statistics

Susceptibilities of each genomospecies to the 16 antibiotics were compared with the entity of all 153 strains by *t*-test (Table 2).

## RESULTS

### Identification and isolation frequencies of new *C. jeikeium* genomospecies

A total of 153 out of 190 isolates included in this study, which were initially identified as *C. jeikeium*, could be finally confirmed by combining MALDI-TOF MS with 16S rRNA gene sequence analysis. Thirty-one out of 141 isolates collected from 1994 to 1999 were identified as distinct *Corynebacterium* spp., i.e., *Corynebacterium tuberculostearicum* (*n* = 17), *Corynebacterium amycolatum* (*n* = 6), *Corynebacterium mucifaciens* (*n* = 3), *Corynebacterium accolens* (*n* = 2), *Corynebacterium afermentans* subsp. *lipophilum* (*n* = 1), *Corynebacterium bovis* (*n* = 1), and *Corynebacterium pilbarense* (*n* = 1); further four strains could not be cultivated any more. Three reference strains, i.e., the *C. jeikeium* type strain (Cj-56, ATCC 43734), B30049-DNA group B (Cj-74), and B15507-DNA group C (Cj-72), were confirmed by MALDI-TOF MS and 16S rRNA gene sequence analysis, whereas the reference strain B6225-DNA group D (Cj-75) was only confirmed by 16S rRNA gene sequence analysis but not by MALDI-TOF MS. Forty-three out of 45 isolates collected between 2012 and 2019 could be confirmed as *C. jeikeium*; one isolate could not be cultivated, and one isolate was contaminated with *Staphylococcus caprae* (Table S1).

**Fig 1:**
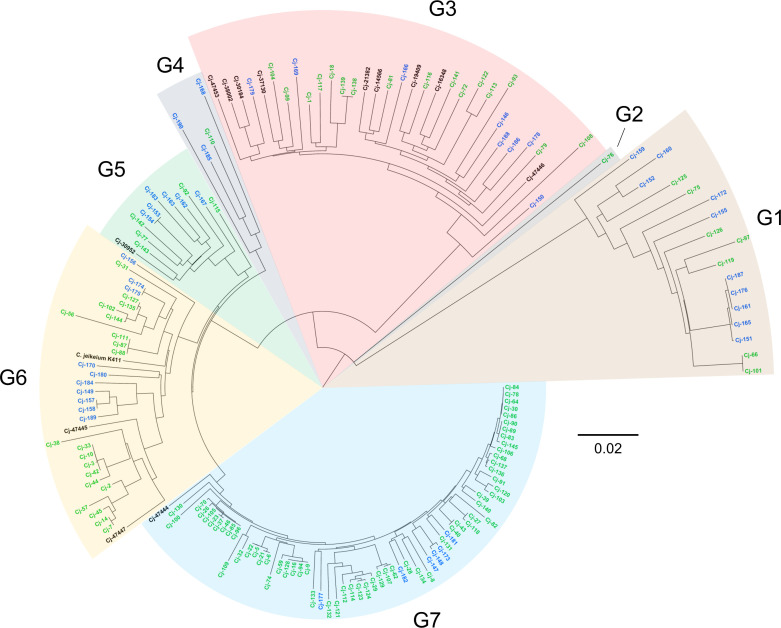
Phylogenetic neighbor-joining tree based on core genome SNPs of the 153 *C*. *jeikeium* isolates reveals seven individual genomospecies. Isolates analyzed in this study are displayed in green (collected 1994–1999) and blue font (collected 2012–2019), respectively; the published genomes of 13 strains from the study of Salipante et al. (23) and of the reference strain K411 (6) are integrated and shown in black font. G1 to G7 represent the seven genomospecies. The scale bar refers to nucleotide substitutions per site.

Core genome SNP-based phylogenetic analysis indicated that the 153 *C*. *jeikeium* strains were distributed among seven clusters (Fig. 1). The dominant cluster 7 encompassed 66 isolates, including strain Cj-74, a representative of *C. jeikeium* DNA group B (13). The cluster 6 with a total of 30 strains represented the second largest clade containing Cj-56, the type strain ATCC 43734 corresponding to the representative of *C. jeikeium* DNA group A (13). Cluster 3 with 24 isolates comprised Cj-72, a representative of *C. jeikeium* DNA group C (13). Clusters 1, 5, and 4 had fewer representatives with 17, 11, and 4 strains, respectively, and cluster 1 included Cj-75, the representative of *C. jeikeium* DNA group D (13). Finally, the isolate *C. jeikeium* 76 (Cj-76) was the sole member of its taxa, referred to as cluster 2 (Fig. 1). Our phylogenetic analysis was augmented by published sequences of isolates from a study by Salipante et al. (23). As observed before, these strains distributed to four different clusters, corresponding to cluster 3 (Cj-47453, Cj-38002, Cj-30184, Cj-37130, Cj-21382, Cj-14566, Cj-19409, Cj-16348, and Cj-47446), cluster 5 (Cj-30952), cluster 6 (Cj-47445, Cj-47447), and cluster 7 (Cj-47444) in our analysis (Fig. 1). *C. jeikeium* K411 described by Tauch et al. (6) was in cluster 6.

We found a high genetic uniformity between the isolates within each cluster (i.e., ANI ≥95%; Table 1). In contrast, pairwise genome comparisons indicated an increased genetic diversity between the different clusters (i.e., ANI <95%; Table 1). Based on these findings, the seven clusters were considered individual genomospecies, respectively (hereafter referred to as G1 to G7). Isolates from the genomospecies G1, G2, and G3 were particularly divergent with an ANI <90%. In contrast, the members of the genomospecies G4, G5, G6, and G7 were more closely related with an ANI between 90% and 95%. Gene prediction and annotation based on the *de novo* sequence assemblies revealed a median of 2011 coding sequences (CDS) and a median genome size of 2.33 Mbp (Table S2).

**TABLE 1:** Whole-genome average nucleotide identity (ANI)^*a*^ reveals seven *C. jeikeium* genomospecies

	Genomospecies						
Genomospecies	1	2	3	4	5	6	7
**1**	**98.64**	85.72	85.14	85.13	84.80	84.98	85.14
**2**	85.72	**100^*b*^**	86.26	88.00	87.92	87.37	88.23
**3**	85.14	86.26	**97.95**	87.34	87.70	87.34	87.64
**4**	85.13	88.00	87.34	**97.97**	93.75	92.19	94.90
**5**	84.80	87.92	87.70	93.75	**98.03**	92.71	94.42
**6**	84.98	87.37	87.34	92.19	92.71	**97.41**	93.20
**7**	85.14	88.23	87.64	94.90	94.42	93.20	**98.03**

^*a*^ The pairwise ANI was computed using the FastANI algorithm (29) and the whole genome sequence data generated for each of the 153 isolates. The matrix depicted the mean pairwise ANI calculated within and between the seven *C*. *jeikeium* clusters. A pairwise ANI value ≥95% indicates the two tested isolates belong to the same species. ANI values of intra-genomospecies comparison are highlighted in bold font.

^*b*^ Single isolate.

### AST of the *C. jeikeium* genomospecies

For antibiotics with a clinical breakpoint, the distributions are indicated with the CBPs in the Supplementary Material (Fig. S1, S3, S4, S6, S7, S9, S13 and S15). For antibiotics without a clinical breakpoint, the provisional ECOFF values separating the WT and non-WT populations are provided, i.e., 25 mm for ceftriaxone, 21 mm for trimethoprim-sulfamethoxazole, 21 mm for tigecycline, 17 mm for teicoplanin, 21 mm for meropenem, 25 mm for imipenem, 23 mm for gentamicin, and 21 mm for fusidic acid (Fig. S2, S5, S8, S10 to S12, S14 and S16).

The seven genomospecies share common susceptibilities and resistances, but the pattern of each genomospecies is typical and has some differences from the others (Table 2). For penicillin (PEN), ceftriaxone (CRO), clindamycin (CLI), erythromycin (ERY), and trimethoprim-sulfamethoxazole (SXT), the 153 strains displayed a unimodal distribution (Table 2; Fig. S1 to S5), i.e., >80% of all isolates exhibited resistance to these five antibiotics. With one exception each, all strains in genomospecies 6 and 7 were resistant to penicillin and ceftriaxone (Table 2; Fig. S1 and S2). Only six out of 153 strains were susceptible to clindamycin (Table 2; Fig. S3). Twenty-four strains were tested susceptible to erythromycin; genomospecies 5 to 7 were more resistant than genomospecies 1 to 4 (Table 2; Fig. S4). Sixteen out of 153 strains were susceptible to trimethoprim-sulfamethoxazole and were found primarily within genomospecies G3 (Table 2; Fig. S5).

**TABLE 2:** Antibiotic susceptibility of genomospecies 1 to 7 to 16 antibiotics

Genomospecies number	Antibiotic	PEN^*a*^	CRO^*b*^	CLI^*c*^	ERY^*d*^	SXT^*b*^	LNZ^*c*^	TET^*c*^	TGC^*b*^	VAN^*c*^	TEC^*b*^	MEM^*b*^	IPM^*b*^	CIP^*a*^	GEN^*b*^	RIF^*c*^	FA^*b*^
G 1	n susceptible	6	10	1	7	3	16	16	16	17	16	11	10	10	8	14	9
*n* = 17	% susceptible	35^*f*^	59^*f*^	5.9	41^*f*^	18	94.1	94.1	94.1^*g*^	100	94.1	65^*f*^	59^*f*^	59^*f*^	47	82	53
G 2	n susceptible	1	1	1	1	1	1	1	1	1	1	1	1	0	1	1	1
*n* = 1^*e*^	% susceptible																
G 3	n susceptible	4	4	1	9	10	23	23	24	24	24	19	17	19	20	23	20
*n* = 24	% susceptible	17	17	4.2	38^*f*^	42^*f*^	95.8	95.8	100	100	100	77^*f*^	71^*f*^	79^*f*^	83^*f*^	95.8^*f*^	83^*f*^
G 4	n susceptible	1	2	1	1	0	4	3	4	4	4	4	4	3	4	2	3
*n* = 4^*e*^	% susceptible																
G 5	n susceptible	2	6	0	1	1	11	9	11	11	11	10	7	9	9	11	11
*n* = 11	% susceptible	18	55^*f*^	0	9.1	9.1	100	82	100	100	100	90.9^*f*^	64^*f*^	82^*f*^	82^*f*^	100	100^*f*^
G 6	n susceptible	1	1	1	2	1	30	24	30	30	30	1	1	2	1	19	14
*n* = 30	% susceptible	3.3	3.3	3.3	6.6	3.3	100	80^*g*^	100	100	100	3.3^*g*^	3.3^*g*^	6.6^*g*^	3.3^*g*^	63	47
G 7	n susceptible	1	1	1	3	0	63	63	66	66	65	4	5	3	7	41	24
*n* = 66	% susceptible	1.5^*g*^	1.5^*g*^	1.5	4.6^*g*^	0^*g*^	95.4	95.4	100	100	98.5	6.1^*g*^	7.5^*g*^	4.6^*g*^	11^*g*^	62	36^*g*^
All	n susceptible	16	25	6	24	16	148	139	152	153	151	50	45	46	50	111	82
*n* = 153	% susceptible	10.5	16.3	3.9	15.7	10.5	96.7	90.8	99.4	100	98.7	32.7	29.4	30.1	33	72.5	53.6

^*a*^ I for susceptible, increased exposure, are calculated as susceptible.

^*b*^ WT population with inhibition zones greater than the eyeball ECOFF are considered to be susceptible.

^*c*^ CPB according EUCAST version 15.

^*d*^ CPB for *C. diphtheriae* and *C. ulcerans* of the EUCAST version 15 was applied.

^*e*^ % susceptibility not indicated due to low absolute numbers (*n* =< 10).

^*f*^ % susceptibility of a genomospecies to the corresponding antibiotic significantly higher than the % susceptibility of all 153 isolates (T-test, *P *<0.05).

^*g*^ % susceptibility of a genomospecies to the corresponding antibiotic significantly lower than the % susceptibility of all 153 isolates (T-test, *P* < 0.05).

In contrast to the aforementioned Antibiotics, the *C. jeikeium* strains showed a unimodal susceptible distribution for linezolid (LNZ), tetracycline (TET), tigecycline (TGC), vancomycin (VAN), and teicoplanin (TEC) (Fig. S6 to S10) and over 90% were susceptible to all five antibiotics (Table 2). All but two isolates were susceptible to vancomycin and teicoplanin (Tables S3 and S9). Furthermore, all but one isolate (Cj-187, inhibition zone diameter of 14 mm) were susceptible against tigecycline (Table S3). Out of the 153 tested isolates, only five were resistant to linezolid, and only 14 isolates were resistant to tetracycline (Tables S3 to S9). Of 153 strains, 132 (86.3%) were susceptible to these five antibiotics.

For meropenem (MEM), imipenem (IPM), ciprofloxacin (CIP), gentamicin (GEN), rifampicin (RIF), and fusidic acid (FA), all 153 isolates displayed susceptibility rates of >20% and resistance rates of >20%, respectively (Fig. S11 to S16). Notably, 27 of the 29 isolates that were resistant to all six antibiotics originated from genomospecies G6 and G7; in addition, 31 of the 33 isolates that were resistant to five of the six antibiotics, yet susceptible to either rifampicin or fusidic acid, also originated from genomospecies G6 and G7 (Tables S8 and S9). Conversely, 26 of the 27 isolates that were susceptible to all six antibiotics were from genomospecies G1, G2, G3, G4, and G5 (Tables S4 to S7).

## DISCUSSION

Before the introduction of MALDI-TOF and gene sequencing-based identification of bacteria in the routine diagnostics of our laboratory, *C. jeikeium* was identified based on different biochemical characteristics and the analysis of fatty acid patterns (3, 7, 34). In the study presented here, re-analysis of isolates collected between 1994 and 1999 revealed frequent incorrect assignment to “*C. jeikeium*” (31 out of 141 isolates), illustrating that conventional methods were associated with difficulties. This was mainly due to incorrect assessment of lipophilism or similar carbohydrate metabolism profiles of various multi-resistant strains, which frequently led to misidentification of lipophilic *C. tuberculostaticum* when its fermentation of fructose was not evaluated (3, 7). In contrast, clinical *C. jeikeium* isolates collected between 2012 and 2019 were mainly identified by MALDI-TOF and re-analysis by 16S rRNA gene sequencing, and repeated MALDI-TOF confirmed the identification. Although MALDI-TOF MS can correctly identify *C. jeikeium*, it is not possible to differentiate the multiresistant strains from the more susceptible strains nor to correlate with genomospecies described by Riegel et al. and Salipante et al. (13, 23). Older extensive biochemical studies in the 1990s at our institution were also unable to identify a phenotypic difference between susceptible and resistant strains (unpublished). The microbiological identification of *C. jeikeium* in cases of serious infections often prompts empirical therapy with glycopeptides until the results of AST become available, typically within 2 days. Thus, faster identification of less-resistant genomospecies could help to adjust the empirical therapy.

The advent of WGS technologies has enabled the in-depth analysis of genomospecies of *C. jeikeium* (23). The findings presented here are in accordance with earlier studies that revealed the extensive genomic diversity of the species *C. jeikeium* (13, 24). These reports suggested the presence of at least four distinct “genomospecies” or “DNA groups”, respectively, which overlap with five of the genomospecies (G1, G3, G5, G6, and G7) found in the analysis of our *C. jeikeium* collection. The latter represents the majority of isolates over the 25-year collection period. The bioinformatic analysis of the genomes of the published 13 strains by Salipante et al. (23) and the reference strain K111 by Tauch et al. (6) matched accurately with our strains. For instance, all nine strains classified as genomospecies 1 by Salipante et al. were found in our G3, and the K111 strain along with two strains classified as genomospecies 2 by Salipante et al. was identified in our G6 (Fig. 1). The identification of the two additional genomospecies G2 and G4, which comprise a total of only one and four isolates, respectively, can possibly be attributed to the much larger number of isolates analyzed as compared to previous studies.

ANI has been shown to be a robust method to assess species boundaries (29, 35, 36). Based on the ANI values obtained by pairwise comparison of their isolates (<95%), Salipante et al. suggested that each of the four *C*. *jeikeium* genomospecies should be considered as a separate species (23). Likewise, considering the threshold value of <95% for species delineation, all seven genomospecies identified in the present study may represent different (sub-)species. G1, G2, and G3 exhibit lower ANI values when compared with each other and to G4–G7 (Table 1), indicating greater genetic divergence. Conversely, G4–G7 display ANI values closer to the species-defining threshold, suggesting they are more closely related to each other. This aligns with the findings of Salipante et al., where their genomospecies 1 (corresponding to our G3) exhibits ANI values below 90% when compared with genomospecies 2, 3, and 4 (corresponding to our G5–G7). Genomospecies 2, 3, and 4 demonstrate ANI values ranging from 91% to 94% when compared with each other (23), similar to the ANI values observed in our comparisons of G5 with G6 and G7 (92.7% and 94.4%, respectively), and between G6 and G7 (93.2%) (Table 2).

The AST of the 153 isolates analyzed in the present study revealed both multi-drug-resistant and susceptible strains, based on EUCAST clinical breakpoints (CBP) for corynebacteria or eyeball ECOFFs if CBPs were not available. It should be emphasized that these eyeball ECOFF values based on inhibition zones do not represent clinical breakpoints but were helpful in classifying the susceptible wild-type population and the resistant non-wild-type population. They serve only to classify strains as susceptible or resistant in order to describe resistance patterns in the various JK groups. MICs from several independent collections are intended for the creation of ECOFFs (33). In contrast, for antibiotics with clinical breakpoints, inhibition zone diameters for classification as susceptible or resistant are established, even if the incubation time for isolates with insufficient growth after 16–20 h incubation is prolonged to 40–44 h (32). Furthermore, in a previous study with 58 isolates of *C. jeikeium*, the disk diffusion test of penicillin, gentamicin, teicoplanin, and vancomycin correlated very well with the agar dilution reference method (37).

In our study, resistance to penicillin, ceftriaxone, and erythromycin was in line with earlier reports (13, 23). The resistance to both clindamycin and trimethoprim-sulfamethoxazole was a common feature for our collection and for that of Salipante et al. (23). Altogether, resistance to penicillin, ceftriaxone, erythromycin, clindamycin, and trimethoprim-sulfamethoxazole was not a marker for a specific genomospecies. Similarly, AST of linezolid, tetracycline, tigecycline, vancomycin, and teicoplanin with a unimodal susceptible distribution did not allow correlating resistance patterns with a specific genomospecies, either.

The resistance towards the other antibiotics, i.e., meropenem, imipenem, ciprofloxacin, gentamicin, rifampicin, and fusidic acid showed the greatest variation in the different genomospecies so that together with the pattern of the unimodal resistant distributions a correlation with the genomospecies is feasible. Resistance to at least seven of nine antibiotics (penicillin, ceftriaxone, erythromycin, meropenem, imipenem, ciprofloxacin, gentamicin, rifampicin, and fusidic acid) together with a clear resistance (i.e., inhibition zone diameters of 6 mm) to clindamycin and trimethoprim-sulfamethoxazole was a hallmark of genomospecies G6 and G7. Therefore, the identification of the genomospecies G6 and G7 predicts multi-resistance, so that glycopeptides are the first therapeutic option.

We conclude that our study of a large library of *C. jeikeium* clinical isolates revealed a more complex and dynamic phylogenetic structure than earlier studies (13, 23). Only a rapid identification of genomospecies G6 and G7 would have a clinical impact. As soon as clear phenotypic traits can be attributed to the resistant genomospecies G6 and G7 by routine diagnostic tests, genomospecies encompassing mainly antibiotic-susceptible strains should be reclassified. WGS could aid in classification/identification of genomospecies; however, this technology is—at least at the moment–usually not part of the diagnostic work-up in routine diagnostic laboratories. We expect that further work may ultimately establish genomospecies-specific metabolic or physical fingerprints with the aim to accelerate the determination under routine diagnostic conditions that would optimize the prediction of adequate antibiotic treatment.

## Data Availability

All data are available in the main text or the supplementary material. Sequence data generated for this study have been submitted to the NCBI Sequence Read Archive (SRA; http://www.ncbi.nlm.nih.gov/sra) under study accession number PRJNA675720. All other relevant data and materials will be provided upon request to fimkamp@imm.uzh.ch. The complete genome sequence from C. jeikeium K411 is available in the NCBI Sequence Read Archive (https://www.ncbi.nlm.nih.gov/nuccore/) under the GenBank accession number CR931997.1. The sequence data generated by Salipante and coworkers (23) used in this study are available at the NCBI Sequence Read Archive (SRA; http://www.ncbi.nlm.nih.gov/sra) under the accession number SRP045192.

## SUPPLEMENTAL MATERIAL

Supplemental figures and tables jcm.00418-25-s0001.docx Results MALDI-TOF and 16S rRNA gene sequencing results for all 153 isolates, genome sizes (as by WGS), AST data and distributions of inhibition zones of tested antibiotics.

ASM does not own the copyrights to Supplemental Material that may be linked to, or accessed through, an article. The authors have granted ASM a non-exclusive, world-wide license to publish the Supplemental Material files. Please contact the corresponding author directly for reuse.
