# Supplementary material for: Antibiotic resistance profiles of seven genomospecies of Corynebacterium jeikeium analyzed by whole genome sequencing
Source: J Clin Microbiol. 2025 Sep 3;63(10):e00418-25. doi: 10.1128/jcm.00418-25 (PMC12505879; doi:10.1128/jcm.00418-25)
Supplement: Supplemental figures and tables — Results MALDI-TOF and 16S rRNA gene sequencing results for all 153 isolates, genome sizes (as by WGS), AST data and distributions of inhibition zones of tested antibiotics. [file jcm.00418-25-s0001.docx]

**Supplemental Material**

**Supplementary Table 1: MALDI-TOF MS and 16S rRNA gene analysis of 186 clinical isolates and 4 reference strains identified as *C. jeikeium*.**

| Key | MALDI-TOF MS analysis | 16S rRNA gene analysis |
| --- | --- | --- |
| 1 | *Corynebacterium jeikeium* | *Corynebacterium jeikeium* |
| 2 | *Corynebacterium jeikeium* | *Corynebacterium jeikeium* |
| 3 | *Corynebacterium jeikeium* | *Corynebacterium jeikeium* |
| 4 | ***not identified*** | *Corynebacterium tuberculostearicum* |
| 5 | *Corynebacterium jeikeium* | *Corynebacterium jeikeium* |
| 6 | *Corynebacterium jeikeium* | *Corynebacterium jeikeium* |
| 7 | *Corynebacterium jeikeium* | *Corynebacterium jeikeium* |
| 8 | *Corynebacterium jeikeium* | *Corynebacterium jeikeium* |
| 9 | *Corynebacterium jeikeium* | *Corynebacterium jeikeium* |
| 10 | *Corynebacterium jeikeium* | *Corynebacterium jeikeium* |
| 11 | *Corynebacterium tuberculostearicum* |  |
| 12 | *Corynebacterium tuberculostearicum* |  |
| 13 | ***no growth*** |  |
| 14 | *Corynebacterium jeikeium* | *Corynebacterium jeikeium* |
| 15 | *Corynebacterium tuberculostearicum* |  |
| 16 | *Corynebacterium jeikeium* | *Corynebacterium jeikeium* |
| 17 | *Corynebacterium accolens* |  |
| 18 | *Corynebacterium jeikeium* | *Corynebacterium jeikeium* |
| 19 | *Corynebacterium afermentans* ssp. *lipophilum* |  |
| 20 | *Corynebacterium tuberculostearicum* |  |
| 21 | *Corynebacterium jeikeium* | *Corynebacterium jeikeium* |
| 22 | *Corynebacterium jeikeium* | *Corynebacterium jeikeium* |
| 23 | *Corynebacterium jeikeium* | *Corynebacterium jeikeium* |
| 24 | *Corynebacterium tuberculostearicum* |  |
| 25 | ***no growth*** |  |
| 26 | *Corynebacterium jeikeium* | *Corynebacterium jeikeium* |
| 27 | *Corynebacterium jeikeium* | *Corynebacterium jeikeium* |
| 28 | *Corynebacterium jeikeium* | *Corynebacterium jeikeium* |
| 29 | *Corynebacterium jeikeium* | *Corynebacterium jeikeium* |
| 30 | *Corynebacterium jeikeium* | *Corynebacterium jeikeium* |
| 31 | *Corynebacterium jeikeium* | *Corynebacterium jeikeium* |
| 32 | *Corynebacterium jeikeium* | *Corynebacterium jeikeium* |
| 33 | *Corynebacterium jeikeium* | *Corynebacterium jeikeium* |
| 34 | *Corynebacterium tuberculostearicum* |  |
| 35 | *Corynebacterium accolens* |  |
| 36 | *Escherichia coli* and  *Corynebacterium tuberculostearicum* |  |
| 37 | *Corynebacterium jeikeium* | *Corynebacterium jeikeium* |
| 38 | *Corynebacterium jeikeium* | *Corynebacterium jeikeium* |
| 39 | *Corynebacterium jeikeium* | *Corynebacterium jeikeium* |
| 40 | *Corynebacterium jeikeium* | *Corynebacterium jeikeium* |
| 41 | ***no growth*** |  |
| 42 | *Corynebacterium jeikeium* | *Corynebacterium jeikeium* |
| 43 | *Corynebacterium jeikeium* | *Corynebacterium jeikeium* |
| 44 | *Corynebacterium jeikeium* | *Corynebacterium jeikeium* |
| 45 | *Corynebacterium jeikeium* | *Corynebacterium jeikeium* |
| 46 | *Corynebacterium jeikeium* | *Corynebacterium jeikeium* |
| 47 | *Corynebacterium tuberculostearicum* |  |
| 48 | *Corynebacterium amycolatum* |  |
| 49 | *Corynebacterium tuberculostearicum* |  |
| 50 | *Corynebacterium tuberculostearicum* |  |
| 51 | *Corynebacterium mucifaciens* |  |
| 52 | *Corynebacterium tuberculostearicum* |  |
| 53 | *Corynebacterium mucifaciens* |  |
| 54 | *Corynebacterium amycolatum* |  |
| 55 | *Corynebacterium bovis* |  |
| 56  ATCC 43734 | *Corynebacterium jeikeium* | *Corynebacterium jeikeium* |
| 57 | *Corynebacterium jeikeium* | *Corynebacterium jeikeium* |
| 58 | *Corynebacterium amycolatum* |  |
| 59 | *Corynebacterium jeikeium* | *Corynebacterium jeikeium* |
| 60 | *Corynebacterium amycolatum* |  |
| 61 | *Corynebacterium amycolatum* |  |
| 62 | *Corynebacterium jeikeium* | *Corynebacterium jeikeium* |
| 63 | *Corynebacterium amycolatum* |  |
| 64 | *Corynebacterium jeikeium* | *Corynebacterium jeikeium* |
| 65 | *Corynebacterium tuberculostearicum* |  |
| 66 | *Corynebacterium jeikeium* | *Corynebacterium jeikeium* |
| 67 | *Corynebacterium tuberculostearicum* |  |
| 68 | *Corynebacterium jeikeium* | *Corynebacterium jeikeium* |
| 69 | *Corynebacterium tuberculostearicum* |  |
| 70 | *Corynebacterium jeikeium* | *Corynebacterium jeikeium* |
| 71 | *Corynebacterium mucifaciens* |  |
| 72  B15507-DNA group C | *Corynebacterium jeikeium* | *Corynebacterium jeikeium* |
| 73 | *Corynebacterium tuberculostearicum* |  |
| 74  B30049-DNA group B | *Corynebacterium jeikeium* | *Corynebacterium jeikeium* |
| 75 B6225-DNA group D | ***not identified*** | *Corynebacterium jeikeium* |
| 76 | ***not identified*** | *Corynebacterium jeikeium* |
| 77 | *Corynebacterium jeikeium* | *Corynebacterium jeikeium* |
| 78 | *Corynebacterium jeikeium* | *Corynebacterium jeikeium* |
| 79 | *Corynebacterium jeikeium* | *Corynebacterium jeikeium* |
| 80 | *Corynebacterium tuberculostearicum* |  |
| 81 | *Corynebacterium jeikeium* | *Corynebacterium jeikeium* |
| 82 | *Corynebacterium jeikeium* | *Corynebacterium jeikeium* |
| 83 | *Corynebacterium jeikeium* | *Corynebacterium jeikeium* |
| 84 | *Corynebacterium jeikeium* | *Corynebacterium jeikeium* |
| 85 | *Corynebacterium jeikeium* | *Corynebacterium jeikeium* |
| 86 | *Corynebacterium jeikeium* | *Corynebacterium jeikeium* |
| 87 | *Corynebacterium jeikeium* | *Corynebacterium jeikeium* |
| 88 | *Corynebacterium jeikeium* | *Corynebacterium jeikeium* |
| 89 | *Corynebacterium jeikeium* | *Corynebacterium jeikeium* |
| 90 | *Corynebacterium jeikeium* | *Corynebacterium jeikeium* |
| 91 | *Corynebacterium jeikeium* | *Corynebacterium jeikeium* |
| 92 | *Corynebacterium jeikeium* | *Corynebacterium jeikeium* |
| 93 | *Corynebacterium jeikeium* | *Corynebacterium jeikeium* |
| 94 | *Corynebacterium jeikeium* | *Corynebacterium jeikeium* |
| 95 | ***no growth*** |  |
| 96 | *Corynebacterium jeikeium* | *Corynebacterium jeikeium* |
| 97 | *Corynebacterium jeikeium* | *Corynebacterium jeikeium* |
| 98 | ***not identified*** | *Corynebacterium pilbarense* |
| 99 | *Corynebacterium jeikeium* | *Corynebacterium jeikeium* |
| 100 | *Corynebacterium jeikeium* | *Corynebacterium jeikeium* |
| 101 | *Corynebacterium jeikeium* | *Corynebacterium jeikeium* |
| 102 | *Corynebacterium jeikeium* | *Corynebacterium jeikeium* |
| 103 | *Corynebacterium jeikeium* | *Corynebacterium jeikeium* |
| 104 | *Corynebacterium jeikeium* | *Corynebacterium jeikeium* |
| 105 | *Corynebacterium jeikeium* | *Corynebacterium jeikeium* |
| 106 | *Corynebacterium jeikeium* | *Corynebacterium jeikeium* |
| 107 | *Corynebacterium jeikeium* | *Corynebacterium jeikeium* |
| 108 | *Corynebacterium jeikeium* | *Corynebacterium jeikeium* |
| 109 | *Corynebacterium jeikeium* | *Corynebacterium jeikeium* |
| 110 | *Corynebacterium jeikeium* | *Corynebacterium jeikeium* |
| 111 | *Corynebacterium jeikeium* | *Corynebacterium jeikeium* |
| 112 | *Corynebacterium jeikeium* | *Corynebacterium jeikeium* |
| 113 | *Corynebacterium jeikeium* | *Corynebacterium jeikeium* |
| 114 | *Corynebacterium jeikeium* | *Corynebacterium jeikeium* |
| 115 | Mixed culture of  *Corynebacterium jeikeium* and  *Corynebacterium urealyticum* | *Corynebacterium jeikeium* |
| 116 | *Corynebacterium jeikeium* | *Corynebacterium jeikeium* |
| 117 | *Corynebacterium jeikeium* | *Corynebacterium jeikeium* |
| 118 | *Corynebacterium jeikeium* | *Corynebacterium jeikeium* |
| 119 | *Corynebacterium jeikeium* | *Corynebacterium jeikeium* |
| 120 | *Corynebacterium jeikeium* | *Corynebacterium jeikeium* |
| 121 | *Corynebacterium jeikeium* | *Corynebacterium jeikeium* |
| 122 | *Corynebacterium jeikeium* | *Corynebacterium jeikeium* |
| 123 | *Corynebacterium jeikeium* | *Corynebacterium jeikeium* |
| 124 | *Corynebacterium jeikeium* | *Corynebacterium jeikeium* |
| 125 | *Corynebacterium jeikeium* | *Corynebacterium jeikeium* |
| 126 | ***not identified*** | *Corynebacterium jeikeium* |
| 127 | *Corynebacterium jeikeium* | *Corynebacterium jeikeium* |
| 128 | *Corynebacterium jeikeium* | *Corynebacterium jeikeium* |
| 129 | *Corynebacterium jeikeium* | *Corynebacterium jeikeium* |
| 130 | *Corynebacterium jeikeium* | *Corynebacterium jeikeium* |
| 131 | *Corynebacterium jeikeium* | *Corynebacterium jeikeium* |
| 132 | *Corynebacterium jeikeium* | *Corynebacterium jeikeium* |
| 133 | *Corynebacterium jeikeium* | *Corynebacterium jeikeium* |
| 134 | *Corynebacterium jeikeium* | *Corynebacterium jeikeium* |
| 135 | *Corynebacterium jeikeium* | *Corynebacterium jeikeium* |
| 136 | *Corynebacterium jeikeium* | *Corynebacterium jeikeium* |
| 137 | *Corynebacterium jeikeium* | *Corynebacterium jeikeium* |
| 138 | *Corynebacterium jeikeium* | *Corynebacterium jeikeium* |
| 139 | *Corynebacterium jeikeium* | *Corynebacterium jeikeium* |
| 140 | *Corynebacterium jeikeium* | *Corynebacterium jeikeium* |
| 141 | *Corynebacterium jeikeium* | *Corynebacterium jeikeium* |
| 142 | *Corynebacterium jeikeium* | *Corynebacterium jeikeium* |
| 143 | *Corynebacterium jeikeium* | *Corynebacterium jeikeium* |
| 144 | *Corynebacterium jeikeium* | *Corynebacterium jeikeium* |
| 145 | *Corynebacterium jeikeium* | *Corynebacterium jeikeium* |
| 146 | *Corynebacterium jeikeium* | *Corynebacterium jeikeium* |
| 147 | *Corynebacterium jeikeium* | *Corynebacterium jeikeium* |
| 148 | *Corynebacterium jeikeium* | *Corynebacterium jeikeium* |
| 149 | *Corynebacterium jeikeium* | *Corynebacterium jeikeium* |
| 150 | *Corynebacterium jeikeium* | *Corynebacterium jeikeium* |
| 151 | ***not identified*** | *Corynebacterium jeikeium* |
| 152 | ***not identified*** | *Corynebacterium jeikeium* |
| 153 | *Corynebacterium jeikeium* | *Corynebacterium jeikeium* |
| 154 | *Corynebacterium jeikeium* | *Corynebacterium jeikeium* |
| 155 | *Corynebacterium jeikeium* | *Corynebacterium jeikeium* |
| 156 | *Corynebacterium jeikeium* | *Corynebacterium jeikeium* |
| 157 | *Corynebacterium jeikeium* | *Corynebacterium jeikeium* |
| 158 | *Corynebacterium jeikeium* | *Corynebacterium jeikeium* |
| 159 | ***not identified*** | *Corynebacterium jeikeium* |
| 160 | ***not identified*** | *Corynebacterium jeikeium* |
| 161 | *Corynebacterium jeikeium* | *Corynebacterium jeikeium* |
| 162 | *Corynebacterium jeikeium* | *Corynebacterium jeikeium* |
| 163 | *Corynebacterium jeikeium* | *Corynebacterium jeikeium* |
| 164 | **no growth** |  |
| 165 | **not identified** | *Corynebacterium jeikeium* |
| 166 | *Corynebacterium jeikeium* | *Corynebacterium jeikeium* |
| 167 | *Corynebacterium jeikeium* | *Corynebacterium jeikeium* |
| 168 | *Corynebacterium jeikeium* | *Corynebacterium jeikeium* |
| 169 | *Corynebacterium jeikeium* | *Corynebacterium jeikeium* |
| 170 | *Corynebacterium jeikeium* | *Corynebacterium jeikeium* |
| 171 | *Staphylococcus caprae* |  |
| 172 | *Corynebacterium jeikeium* | *Corynebacterium jeikeium* |
| 173 | *Corynebacterium jeikeium* | *Corynebacterium jeikeium* |
| 174 | *Corynebacterium jeikeium* | *Corynebacterium jeikeium* |
| 175 | *Corynebacterium jeikeium* | *Corynebacterium jeikeium* |
| 176 | **not identified** | *Corynebacterium jeikeium* |
| 177 | *Corynebacterium jeikeium* | *Corynebacterium jeikeium* |
| 178 | *Corynebacterium jeikeium* | *Corynebacterium jeikeium* |
| 179 | *Corynebacterium jeikeium* | *Corynebacterium jeikeium* |
| 180 | *Corynebacterium jeikeium* | *Corynebacterium jeikeium* |
| 181 | *Corynebacterium jeikeium* | *Corynebacterium jeikeium* |
| 182 | *Corynebacterium jeikeium* | *Corynebacterium jeikeium* |
| 183 | *Corynebacterium jeikeium* | *Corynebacterium jeikeium* |
| 184 | *Corynebacterium jeikeium* | *Corynebacterium jeikeium* |
| 185 | *Corynebacterium jeikeium* | *Corynebacterium jeikeium* |
| 186 | *Corynebacterium jeikeium* | *Corynebacterium jeikeium* |
| 187 | **not identified** | *Corynebacterium jeikeium* |
| 188 | *Corynebacterium jeikeium* | *Corynebacterium jeikeium* |
| 189 | *Corynebacterium jeikeium* | *Corynebacterium jeikeium* |
| 190 | *Corynebacterium jeikeium* | *Corynebacterium jeikeium* |

**Supplementary Table 2: Predicted Content of coding sequences of the 7 *C. jeikeium* genomospecies**

**Isolates Cluster 1**

| **Time period** | **Isolate** | **Number of genes  (CDS)** | **Genome size**  **(bp)** |
| --- | --- | --- | --- |
| 1994 - 1999 | Cj-66 | 2064 | 2325638 |
|  | Cj-75  B6225-DNA group D | 2033 | 2332949 |
|  | Cj-97 | 2013 | 2303900 |
|  | Cj-101 | 2027 | 2290736 |
|  | Cj-119 | 2024 | 2332774 |
|  | Cj-125 | 2032 | 2352828 |
|  | Cj-126 | 1989 | 2260692 |
| 2012-2019 | Cj-151 | 1984 | 2274189 |
|  | Cj-152 | 1958 | 2239555 |
|  | Cj-155 | 2047 | 2343891 |
|  | Cj-159 | 2750* | 2819520* |
|  | Cj-160 | 2013 | 2270716 |
|  | Cj-161 | 1994 | 2283105 |
|  | Cj-165 | 1998 | 2281305 |
|  | Cj-172 | 2091 | 2351865 |
|  | Cj-176 | 2035 | 2311204 |
|  | Cj-187 | 2036 | 2314410 |

* outlier

**Isolates Cluster 2**

| **Time period** | **Isolate** | **Number of genes  (CDS)** | **Genome size**  **(bp)** |
| --- | --- | --- | --- |
| 1994 - 1999 | Cj-76 | 1977 | 2281121 |

**Isolates Cluster 3**

| **Time period** | **Isolate** | **Number of genes  (CDS)** | **Genome size**  **(bp)** |
| --- | --- | --- | --- |
| 1994 - 1999 | Cj-1 | 1960 | 2269697 |
|  | Cj-18 | 2018 | 2337242 |
|  | Cj-72  B15507-DNA group C | 1973 | 2267065 |
|  | Cj-79 | 1994 | 2298647 |
|  | Cj-81 | 1967 | 2289747 |
|  | Cj-93 | 1982 | 2292009 |
|  | Cj-99 | 1957 | 2261323 |
|  | Cj-104 | 1931 | 2239375 |
|  | Cj-108 | 1948 | 2245755 |
|  | Cj-113 | 1969 | 2266083 |
|  | Cj-116 | 2008 | 2315045 |
|  | Cj-117 | 1940 | 2238885 |
|  | Cj-122 | 1949 | 2254069 |
|  | Cj-138 | 1999 | 2309840 |
|  | Cj-139 | 1996 | 2312051 |
|  | Cj-141 | 2051 | 2352050 |
| 2012-2019 | Cj-146 | 1985 | 2293462 |
|  | Cj-150 | 1992 | 2309506 |
|  | Cj-166 | 2058 | 2337661 |
|  | Cj-169 | 1985 | 2290026 |
|  | Cj-178 | 1979 | 2292211 |
|  | Cj-179 | 1955 | 2278136 |
|  | Cj-186 | 1934 | 2242031 |
|  | Cj-188 | 1973 | 2314438 |

**Isolates Cluster 4**

| **Time period** | **Isolate** | **Number of genes  (CDS)** | **Genome size**  **(bp)** |
| --- | --- | --- | --- |
| 1994 - 1999 | Cj-110 | 1994 | 2315177 |
| 2012-2019 | Cj-168 | 2014 | 2322680 |
|  | Cj-185 | 1930 | 2242866 |
|  | Cj-190 | 1988 | 2263793 |

**Isolates Cluster 5**

| **Time period** | **Isolate** | **Number of genes  (CDS)** | **Genome size**  **(bp)** |
| --- | --- | --- | --- |
| 1994 - 1999 | Cj-77 | 1974 | 2304982 |
|  | Cj-92 | 1953 | 2264503 |
|  | Cj-115 | 2270* | 2355680* |
|  | Cj-142 | 2035 | 2349862 |
|  | Cj-143 | 1943 | 2293067 |
| 2012-2019 | Cj-153 | 1992 | 2300033 |
|  | Cj-154 | 1932 | 2253249 |
|  | Cj-162 | 1956 | 2299805 |
|  | Cj-163 | 2001 | 2302734 |
|  | Cj-167 | 2573* | 3113878* |
|  | Cj-183 | 2002 | 2340726 |

* outlier

**Isolates Cluster 6**

| **Time period** | **Isolate** | **Number of genes  (CDS)** | **Genome size**  **(bp)** |
| --- | --- | --- | --- |
| 1994 - 1999 | Cj-2 | 1999 | 2316494 |
|  | Cj-3 | 2088 | 2415424 |
|  | Cj-7 | 2052 | 2361972 |
|  | Cj-10 | 2095 | 2413978 |
|  | Cj-14 | 2052 | 2362513 |
| 1994 - 1999 | Cj-31 | 2080 | 2410577 |
|  | Cj-33 | 2089 | 2411677 |
|  | Cj-38 | 1991 | 2311019 |
|  | Cj-42 | 2090 | 2410970 |
|  | Cj-44 | 2073 | 2393445 |
|  | Cj-45 | 2059 | 2373607 |
|  | Cj-56  ATCC 43734 | 2139 | 2436211 |
|  | Cj-57 | 2073 | 2405880 |
|  | Cj-87 | 1998 | 2322498 |
|  | Cj-88 | 1996 | 2322807 |
|  | Cj-102 | 2037 | 2358123 |
|  | Cj-111 | 1995 | 2324785 |
|  | Cj-127 | 2028 | 2347344 |
|  | Cj-135 | 2030 | 2348241 |
|  | Cj-144 | 2033 | 2354959 |
| 2012-2019 | Cj-149 | 2001 | 2318216 |
|  | Cj-156 | 2685* | 3125925* |
|  | Cj-157 | 2098 | 2426874 |
|  | Cj-158 | 2081 | 2412884 |
|  | Cj-170 | 2052 | 2371055 |
|  | Cj-174 | 1985 | 2309154 |
|  | Cj-175 | 1984 | 2309248 |
|  | Cj-180 | 2083 | 2393303 |
|  | Cj-184 | 2041 | 2358281 |
|  | Cj-189 | 2071 | 2409375 |

* outlier

**Isolates Cluster 7**

| **Time period** | **Isolate** | **Number of genes  (CDS)** | **Genome size**  **(bp)** |
| --- | --- | --- | --- |
| 1994 - 1999 | Cj-5 | 2137 | 2481141 |
|  | Cj-6 | 1974 | 2302505 |
|  | Cj-8 | 2054 | 2372629 |
|  | Cj-9 | 2036 | 2360852 |
|  | Cj-16 | 2037 | 2362056 |
| 1994 - 1999 | Cj-21 | 1975 | 2303336 |
|  | Cj-22 | 1989 | 2308295 |
|  | Cj-23 | 2022 | 2349191 |
|  | Cj-26 | 2735* | 3051302* |
|  | Cj-27 | 1968 | 2290519 |
|  | Cj-28 | 2066 | 2359287 |
|  | Cj-29 | 1958 | 2297658 |
|  | Cj-30 | 2002 | 2321854 |
|  | Cj-32 | 2576* | 2920354* |
|  | Cj-37 | 2022 | 2349810 |
|  | Cj-39 | 1961 | 2285496 |
|  | Cj-40 | 2014 | 2342532 |
|  | Cj-43 | 2015 | 2342065 |
|  | Cj-46 | 2103 | 2429599 |
|  | Cj-59 | 2042 | 2365209 |
|  | Cj-62 | 1986 | 2323424 |
|  | Cj-64 | 2001 | 2322037 |
|  | Cj-68 | 1968 | 2295434 |
|  | Cj-70 | 2100 | 2420904 |
|  | Cj-74  B30049-DNA group B | 2008 | 2353785 |
|  | Cj-78 | 2004 | 2322529 |
|  | Cj-82 | 1950 | 2272472 |
|  | Cj-83 | 1996 | 2321626 |
|  | Cj-84 | 2000 | 2321847 |
|  | Cj-85 | 2127 | 2446197 |
|  | Cj-86 | 2003 | 2321888 |
|  | Cj-89 | 2004 | 2321721 |
|  | Cj-90 | 2005 | 2321550 |
|  | Cj-91 | 2026 | 2353105 |
|  | Cj-94 | 2032 | 2356243 |
|  | Cj-96 | 2111 | 2430504 |
|  | Cj-100 | 2025 | 2338782 |
|  | Cj-103 | 2065 | 2392136 |
|  | Cj-105 | 2110 | 2432237 |
| 1994 - 1999 | Cj-106 | 2000 | 2314185 |
|  | Cj-107 | 1959 | 2286182 |
|  | Cj-109 | 1967 | 2295620 |
|  | Cj-112 | 1957 | 2306089 |
|  | Cj-114 | 1989 | 2317559 |
|  | Cj-118 | 1965 | 2282843 |
|  | Cj-120 | 2067 | 2399474 |
|  | Cj-121 | 1980 | 2311503 |
|  | Cj-123 | 1944 | 2278786 |
|  | Cj-124 | 1943 | 2278932 |
|  | Cj-128 | 1971 | 2293075 |
|  | Cj-129 | 1958 | 2285481 |
|  | Cj-130 | 2030 | 2373216 |
|  | Cj-131 | 1981 | 2299461 |
|  | Cj-132 | 1998 | 2339147 |
|  | Cj-133 | 2076 | 2413525 |
|  | Cj-134 | 1992 | 2308780 |
|  | Cj-136 | 2021 | 2352316 |
|  | Cj-137 | 2027 | 2354675 |
|  | Cj-140 | 1995 | 2330065 |
|  | Cj-145 | 1986 | 2298938 |
| 2012-2019 | Cj-147 | 2020 | 2356648 |
|  | Cj-148 | 1999 | 2343806 |
|  | Cj-173 | 2446* | 2953666* |
|  | Cj-177 | 2001 | 2325792 |
|  | Cj-181 | 2012 | 2353996 |
|  | Cj-182 | 2004 | 2327023 |

* outlier

**Supplementary Table 3: Antibiotic susceptibility (green) and resistance (red) of genomospecies 1 with inhibition zones (mm)**

| Time period | Isolate Nr. | PEN | CRO | CLI | ERY | SXT | LNZ | TET | TGC | VAN | TEC | MEM | IPM | CIP | GEN | RIF | FA |
| --- | --- | --- | --- | --- | --- | --- | --- | --- | --- | --- | --- | --- | --- | --- | --- | --- | --- |
| 1994 -1999 | 66 | 24 | 30 | 6 | 17 | 30 | 28 | 40 | 28 | 26 | 40 | 40 | 31 | 32 | 24 | 40 | 34 |
|  | 75 | 6 | 32 | 6 | 11 | 17 | 40 | 34 | 30 | 25 | 24 | 38 | 40 | 32 | 24 | 40 | 20 |
|  | 97 | 16 | 30 | 6 | 35 | 19 | 38 | 36 | 32 | 26 | 11 | 40 | 40 | 36 | 24 | 40 | 40 |
|  | 101 | 26 | 32 | 10 | 15 | 28 | 40 | 35 | 32 | 25 | 23 | 40 | 26 | 30 | 30 | 38 | 40 |
|  | 119 | 6 | 6 | 22 | 36 | 6 | 18 | 36 | 30 | 24 | 22 | 6 | 6 | 6 | 30 | 39 | 28 |
|  | 125 | 22 | 30 | 16 | 30 | 6 | 34 | 36 | 30 | 24 | 22 | 36 | 40 | 30 | 27 | 34 | 34 |
|  | 126 | 6 | 26 | 10 | 34 | 9 | 40 | 34 | 28 | 23 | 21 | 32 | 36 | 30 | 32 | 40 | 30 |
| 2012  -2019 | 151 | 6 | 6 | 6 | 6 | 17 | 40 | 40 | 28 | 28 | 26 | 6 | 6 | 6 | 9 | 40 | 17 |
|  | 152 | 6 | 28 | 10 | 36 | 14 | 36 | 34 | 32 | 22 | 22 | 40 | 40 | 32 | 26 | 40 | 40 |
|  | 155 | 6 | 27 | 18 | 30 | 11 | 32 | 30 | 27 | 24 | 22 | 32 | 40 | 28 | 22 | 40 | 26 |
|  | 159 | 15 | 30 | 6 | 10 | 24 | 30 | 14 | 30 | 21 | 21 | 34 | 40 | 6 | 19 | 40 | 20 |
|  | 160 | 26 | 32 | 6 | 34 | 17 | 40 | 39 | 30 | 23 | 22 | 40 | 40 | 30 | 20 | 40 | 40 |
|  | 161 | 6 | 6 | 6 | 6 | 6 | 40 | 36 | 32 | 24 | 22 | 6 | 6 | 6 | 6 | 6 | 15 |
|  | 165 | 6 | 6 | 6 | 6 | 15 | 38 | 38 | 30 | 25 | 24 | 12 | 6 | 6 | 6 | 40 | 17 |
|  | 172 | 6 | 6 | 6 | 22 | 16 | 28 | 28 | 24 | 21 | 20 | 6 | 6 | 26 | 22 | 34 | 12 |
|  | 176 | 6 | 19 | 6 | 6 | 13 | 36 | 37 | 31 | 25 | 25 | 22 | 22 | 6 | 6 | 6 | 19 |
|  | 187 | 6 | 6 | 6 | 6 | 12 | 30 | 32 | 14 | 25 | 24 | 14 | 6 | 6 | 10 | 6 | 13 |

**Supplementary Table 4: Antibiotic susceptibility (green) and resistance (red) of genomospecies 2 with inhibition zones (mm)**

| Time period | Isolate Nr. | PEN | CRO | CLI | ERY | SXT | LNZ | TET | TGC | VAN | TEC | MEM | IPM | CIP | GEN | RIF | FA |
| --- | --- | --- | --- | --- | --- | --- | --- | --- | --- | --- | --- | --- | --- | --- | --- | --- | --- |
| 1994  -1999 | 76 | 20 | 40 | 40 | 40 | 40 | 30 | 40 | 40 | 40 | 40 | 40 | 40 | 21 | 26 | 38 | 40 |

**Supplementary Table 5: Antibiotic susceptibility (green) and resistance (red) of genomospecies 3 with inhibition zones (mm)**

| Time  period | Isolate Nr. | PEN | CRO | CLI | ERY | SXT | LNZ | TET | TGC | VAN | TEC | MEM | IPM | CIP | GEN | RIF | FA |
| --- | --- | --- | --- | --- | --- | --- | --- | --- | --- | --- | --- | --- | --- | --- | --- | --- | --- |
| 1994  -1999 | 1 | 6 | 18 | 11 | 31 | 15 | 32 | 31 | 28 | 20 | 19 | 25 | 26 | 25 | 22 | 33 | 27 |
|  | 18 | 6 | 16 | 6 | 6 | 23 | 40 | 33 | 28 | 23 | 23 | 30 | 30 | 6 | 6 | 40 | 28 |
|  | 72 | 17 | 28 | 17 | 40 | 26 | 38 | 39 | 34 | 24 | 22 | 34 | 40 | 40 | 26 | 40 | 34 |
|  | 79 | 6 | 21 | 15 | 15 | 24 | 40 | 40 | 34 | 25 | 23 | 32 | 38 | 36 | 26 | 40 | 32 |
|  | 81 | 6 | 23 | 10 | 40 | 32 | 40 | 40 | 36 | 24 | 22 | 36 | 30 | 34 | 28 | 40 | 31 |
|  | 93 | 6 | 24 | 11 | 35 | 18 | 34 | 36 | 30 | 23 | 21 | 34 | 32 | 30 | 24 | 6 | 30 |
|  | 99 | 6 | 22 | 10 | 34 | 20 | 40 | 36 | 30 | 22 | 20 | 32 | 35 | 32 | 30 | 40 | 30 |
|  | 104 | 6 | 19 | 20 | 34 | 14 | 36 | 35 | 32 | 22 | 22 | 30 | 34 | 30 | 24 | 40 | 32 |
|  | 108 | 16 | 28 | 19 | 32 | 12 | 28 | 32 | 28 | 22 | 22 | 36 | 40 | 30 | 28 | 36 | 28 |
|  | 113 | 12 | 22 | 6 | 10 | 16 | 36 | 24 | 23 | 22 | 20 | 28 | 35 | 30 | 27 | 37 | 28 |
|  | 116 | 6 | 22 | 6 | 22 | 16 | 40 | 40 | 32 | 22 | 22 | 34 | 20 | 34 | 28 | 38 | 30 |
|  | 117 | 16 | 20 | 12 | 35 | 25 | 36 | 38 | 28 | 24 | 21 | 34 | 39 | 34 | 28 | 40 | 30 |
|  | 122 | 9 | 23 | 6 | 13 | 18 | 34 | 35 | 30 | 23 | 20 | 32 | 36 | 28 | 25 | 38 | 28 |
|  | 138 | 6 | 16 | 6 | 6 | 28 | 40 | 32 | 30 | 24 | 24 | 24 | 36 | 6 | 24 | 40 | 30 |
|  | 139 | 6 | 16 | 6 | 6 | 27 | 40 | 32 | 30 | 23 | 21 | 25 | 6 | 6 | 24 | 40 | 30 |
|  | 141 | 6 | 40 | 6 | 16 | 15 | 38 | 34 | 32 | 22 | 23 | 32 | 38 | 36 | 28 | 38 | 30 |
| 2012  -2019 | 146 | 10 | 24 | 6 | 8 | 17 | 24 | 38 | 32 | 25 | 22 | 34 | 40 | 30 | 32 | 40 | 14 |
|  | 150 | 6 | 6 | 6 | 12 | 13 | 36 | 30 | 24 | 24 | 21 | 6 | 6 | 6 | 26 | 38 | 15 |
|  | 166 | 6 | 6 | 6 | 21 | 16 | 36 | 35 | 32 | 24 | 21 | 18 | 12 | 6 | 24 | 40 | 26 |
|  | 169 | 8 | 22 | 14 | 34 | 22 | 37 | 36 | 30 | 23 | 22 | 28 | 36 | 30 | 22 | 38 | 28 |
|  | 178 | 6 | 10 | 6 | 6 | 24 | 40 | 21 | 30 | 23 | 21 | 6 | 6 | 28 | 25 | 38 | 18 |
|  | 179 | 6 | 6 | 6 | 18 | 25 | 40 | 40 | 33 | 26 | 24 | 6 | 6 | 31 | 26 | 40 | 38 |
|  | 186 | 10 | 26 | 6 | 15 | 20 | 36 | 40 | 34 | 26 | 22 | 32 | 40 | 32 | 13 | 40 | 16 |
|  | 188 | 6 | 8 | 6 | 14 | 15 | 34 | 38 | 32 | 24 | 22 | 6 | 6 | 30 | 24 | 40 | 25 |

**Supplementary Table 6: Antibiotic susceptibility (green) and resistance (red) of genomospecies 4 with inhibition zones (mm)**

| Time  period | Isolate Nr. | PEN | CRO | CLI | ERY | SXT | LNZ | TET | TGC | VAN | TEC | MEM | IPM | CIP | GEN | RIF | FA |
| --- | --- | --- | --- | --- | --- | --- | --- | --- | --- | --- | --- | --- | --- | --- | --- | --- | --- |
| 1994  -1999 | 110 | 6 | 17 | 6 | 6 | 6 | 40 | 14 | 26 | 22 | 20 | 40 | 40 | 32 | 27 | 34 | 32 |
| 2012  -2019 | 168 | 6 | 24 | 13 | 13 | 6 | 35 | 33 | 28 | 25 | 23 | 38 | 36 | 6 | 33 | 6 | 28 |
|  | 185 | 6 | 30 | 6 | 6 | 6 | 28 | 33 | 30 | 23 | 24 | 36 | 38 | 28 | 24 | 36 | 22 |
|  | 190 | 22 | 34 | 22 | 26 | 11 | 40 | 30 | 28 | 22 | 20 | 34 | 40 | 28 | 24 | 6 | 16 |

**Supplementary Table 7: Antibiotic susceptibility (green) and resistance (red) of genomospecies 5 with inhibition zones (mm)**

| Time  period | Isolate Nr. | PEN | CRO | CLI | ERY | SXT | LNZ | TET | TGC | VAN | TEC | MEM | IPM | CIP | GEN | RIF | FA |
| --- | --- | --- | --- | --- | --- | --- | --- | --- | --- | --- | --- | --- | --- | --- | --- | --- | --- |
| 1994  -1999 | 77 | 13 | 22 | 6 | 18 | 22 | 40 | 40 | 34 | 26 | 22 | 25 | 30 | 40 | 34 | 40 | 31 |
|  | 92 | 8 | 28 | 6 | 38 | 6 | 40 | 15 | 35 | 25 | 21 | 40 | 40 | 34 | 30 | 40 | 36 |
|  | 115 | 6 | 30 | 6 | 19 | 18 | 40 | 40 | 38 | 26 | 22 | 38 | 36 | 29 | 32 | 40 | 34 |
|  | 142 | 20 | 30 | 6 | 10 | 14 | 36 | 39 | 30 | 23 | 20 | 38 | 40 | 29 | 29 | 40 | 31 |
|  | 143 | 6 | 26 | 6 | 15 | 14 | 31 | 37 | 32 | 22 | 20 | 34 | 34 | 28 | 26 | 36 | 29 |
| 2012  -2019 | 153 | 6 | 18 | 6 | 12 | 6 | 36 | 36 | 28 | 24 | 21 | 32 | 20 | 28 | 28 | 38 | 30 |
|  | 154 | 6 | 18 | 6 | 12 | 6 | 32 | 37 | 26 | 21 | 19 | 28 | 6 | 23 | 25 | 34 | 28 |
|  | 162 | 6 | 6 | 6 | 6 | 15 | 28 | 30 | 23 | 22 | 20 | 6 | 6 | 26 | 22 | 34 | 23 |
|  | 163 | 6 | 26 | 14 | 12 | 6 | 36 | 36 | 32 | 26 | 22 | 36 | 36 | 30 | 28 | 40 | 26 |
|  | 167 | 6 | 20 | 6 | 9 | 6 | 28 | 15 | 26 | 22 | 21 | 26 | 6 | 6 | 13 | 32 | 22 |
|  | 183 | 6 | 28 | 6 | 13 | 9 | 34 | 40 | 32 | 24 | 23 | 34 | 40 | 29 | 26 | 40 | 32 |

**Supplementary Table 8: Antibiotic susceptibility (green) and resistance (red) of genomospecies 6 with inhibition zones (mm)**

| Time  period | Isolate Nr. | PEN | CRO | CLI | ERY | SXT | LNZ | TET | TGC | VAN | TEC | MEM | IPM | CIP | GEN | RIF | FA |
| --- | --- | --- | --- | --- | --- | --- | --- | --- | --- | --- | --- | --- | --- | --- | --- | --- | --- |
| 1994  1999 | 2 | 6 | 6 | 6 | 19 | 6 | 40 | 12 | 30 | 26 | 20 | 6 | 6 | 6 | 6 | 38 | 15 |
|  | 3 | 6 | 6 | 6 | 18 | 6 | 40 | 38 | 36 | 25 | 22 | 6 | 6 | 6 | 6 | 40 | 29 |
|  | 7 | 6 | 6 | 6 | 14 | 6 | 38 | 24 | 32 | 22 | 20 | 6 | 6 | 6 | 6 | 36 | 24 |
|  | 10 | 6 | 6 | 6 | 18 | 6 | 40 | 40 | 35 | 24 | 20 | 6 | 6 | 6 | 6 | 40 | 32 |
|  | 14 | 6 | 6 | 6 | 17 | 6 | 40 | 24 | 34 | 23 | 21 | 6 | 6 | 6 | 6 | 35 | 28 |
|  | 31 | 6 | 9 | 6 | 17 | 6 | 40 | 29 | 31 | 23 | 23 | 13 | 6 | 6 | 6 | 40 | 18 |
|  | 33 | 6 | 6 | 6 | 6 | 6 | 40 | 40 | 34 | 26 | 23 | 6 | 6 | 6 | 6 | 40 | 32 |
|  | 38 | 6 | 6 | 6 | 12 | 6 | 40 | 31 | 23 | 27 | 22 | 6 | 6 | 6 | 6 | 40 | 30 |
|  | 42 | 6 | 6 | 6 | 13 | 6 | 32 | 40 | 31 | 24 | 22 | 6 | 6 | 6 | 6 | 40 | 28 |
|  | 44 | 23 | 40 | 35 | 40 | 40 | 31 | 40 | 38 | 31 | 37 | 40 | 35 | 39 | 40 | 40 | 35 |
|  | 45 | 6 | 6 | 6 | 12 | 6 | 40 | 10 | 31 | 24 | 22 | 6 | 6 | 6 | 6 | 40 | 32 |
|  | 56 | 6 | 15 | 6 | 25 | 6 | 38 | 10 | 24 | 25 | 23 | 18 | 6 | 32 | 6 | 40 | 34 |
|  | 57 | 6 | 6 | 6 | 14 | 6 | 32 | 15 | 23 | 26 | 24 | 6 | 6 | 6 | 6 | 40 | 22 |
|  | 87 | 6 | 6 | 6 | 10 | 6 | 40 | 34 | 31 | 26 | 22 | 6 | 6 | 6 | 6 | 6 | 16 |
|  | 88 | 6 | 6 | 6 | 12 | 6 | 36 | 36 | 34 | 25 | 22 | 6 | 6 | 6 | 6 | 6 | 17 |
|  | 102 | 6 | 6 | 6 | 6 | 6 | 36 | 26 | 30 | 23 | 21 | 6 | 6 | 6 | 6 | 36 | 10 |
|  | 111 | 6 | 6 | 6 | 10 | 6 | 40 | 38 | 30 | 25 | 22 | 6 | 7 | 6 | 6 | 6 | 16 |
|  | 127 | 6 | 6 | 6 | 6 | 6 | 36 | 30 | 33 | 25 | 21 | 6 | 6 | 6 | 6 | 10 | 19 |
|  | 135 | 6 | 6 | 6 | 6 | 6 | 39 | 30 | 30 | 24 | 20 | 6 | 6 | 6 | 6 | 10 | 12 |
|  | 144 | 6 | 7 | 6 | 6 | 6 | 40 | 30 | 34 | 25 | 22 | 7 | 6 | 6 | 6 | 12 | 14 |
| 2012  -2019 | 149 | 6 | 6 | 6 | 14 | 6 | 38 | 37 | 34 | 22 | 20 | 6 | 6 | 6 | 6 | 32 | 30 |
|  | 156 | 6 | 6 | 6 | 6 | 6 | 36 | 10 | 37 | 21 | 20 | 6 | 6 | 6 | 6 | 6 | 13 |
|  | 157 | 6 | 6 | 6 | 6 | 6 | 32 | 35 | 35 | 21 | 21 | 6 | 7 | 6 | 6 | 39 | 26 |
|  | 158 | 6 | 6 | 6 | 16 | 6 | 38 | 39 | 31 | 24 | 22 | 6 | 6 | 6 | 6 | 40 | 30 |
|  | 170 | 6 | 6 | 6 | 6 | 6 | 35 | 20 | 32 | 24 | 21 | 6 | 6 | 6 | 6 | 6 | 16 |
| Time  period | Isolate Nr. | PEN | CRO | CLI | ERY | SXT | LNZ | TET | TGC | VAN | TEC | MEM | IPM | CIP | GEN | RIF | FA |
| 2012  -2019 | 174 | 6 | 6 | 6 | 6 | 6 | 36 | 33 | 29 | 21 | 21 | 6 | 6 | 6 | 6 | 6 | 12 |
|  | 175 | 6 | 6 | 9 | 6 | 6 | 36 | 32 | 28 | 22 | 20 | 6 | 6 | 6 | 7 | 7 | 12 |
|  | 180 | 6 | 6 | 6 | 6 | 6 | 40 | 28 | 27 | 24 | 22 | 6 | 6 | 6 | 6 | 6 | 18 |
|  | 184 | 6 | 6 | 6 | 15 | 6 | 36 | 33 | 35 | 24 | 23 | 6 | 6 | 6 | 6 | 39 | 19 |
|  | 189 | 6 | 6 | 6 | 15 | 6 | 38 | 32 | 38 | 24 | 21 | 6 | 6 | 6 | 6 | 40 | 17 |

**Supplementary Table 9: Antibiotic susceptibility (green) and resistance (red) of cluster 7 with inhibition zones (mm)**

| Time  period | Isolate Nr. | PEN | CRO | CLI | ERY | SXT | LNZ | TET | TGC | VAN | TEC | MEM | IPM | CIP | GEN | RIF | FA |
| --- | --- | --- | --- | --- | --- | --- | --- | --- | --- | --- | --- | --- | --- | --- | --- | --- | --- |
| 1994  -1999 | 5 | 6 | 6 | 6 | 6 | 6 | 32 | 37 | 36 | 26 | 23 | 6 | 6 | 6 | 6 |  | 12 |
|  | 6 | 6 | 6 | 6 | 24 | 6 | 40 | 36 | 36 | 28 | 24 | 6 | 6 | 6 | 6 | 6 | 16 |
|  | 8 | 40 | 40 | 40 | 16 | 8 | 24 | 40 | 40 | 37 | 16 | 40 | 37 | 27 | 40 | 25 | 14 |
|  | 9 | 6 | 6 | 6 | 6 | 6 | 28 | 26 | 26 | 22 | 20 | 6 | 6 | 6 | 6 | 6 | 12 |
|  | 16 | 6 | 6 | 6 | 6 | 6 | 40 | 37 | 32 | 28 | 23 | 6 | 6 | 6 | 6 | 6 | 14 |
|  | 21 | 6 | 6 | 6 | 6 | 6 | 38 | 36 | 32 | 27 | 24 | 6 | 6 | 6 | 6 | 6 | 16 |
|  | 22 | 6 | 6 | 6 | 6 | 7 | 40 | 39 | 32 | 21 | 24 | 6 | 6 | 6 | 6 | 6 | 14 |
|  | 23 | 6 | 6 | 6 | 6 | 6 | 40 | 39 | 38 | 28 | 24 | 6 | 6 | 6 | 6 | 6 | 17 |
|  | 26 | 6 | 6 | 6 | 6 | 6 | 21 | 38 | 34 | 22 | 22 | 12 | 6 | 6 | 6 | 7 | 19 |
|  | 27 | 6 | 6 | 6 | 6 | 6 | 40 | 34 | 30 | 29 | 40 | 6 | 6 | 6 | 6 | 6 | 25 |
|  | 28 | 6 | 6 | 6 | 6 | 6 | 40 | 40 | 34 | 28 | 23 | 12 | 6 | 6 | 6 | 6 | 32 |
|  | 29 | 6 | 6 | 6 | 6 | 6 | 38 | 40 | 32 | 28 | 25 | 6 | 6 | 6 | 6 | 40 | 32 |
|  | 30 | 6 | 6 | 6 | 6 | 6 | 40 | 28 | 26 | 29 | 28 | 6 | 6 | 6 | 6 | 40 | 13 |
|  | 32 | 6 | 10 | 6 | 6 | 6 | 40 | 30 | 28 | 26 | 25 | 16 | 6 | 6 | 6 | 18 | 22 |
|  | 37 | 6 | 6 | 6 | 6 | 6 | 20 | 40 | 35 | 25 | 22 | 6 | 6 | 6 | 6 | 10 | 18 |
|  | 39 | 6 | 10 | 6 | 6 | 6 | 34 | 31 | 26 | 28 | 24 | 15 | 18 | 27 | 6 | 40 | 18 |
|  | 40 | 6 | 6 | 6 | 6 | 6 | 36 | 38 | 32 | 28 | 32 | 6 | 6 | 6 | 6 | 40 | 17 |
|  | 43 | 6 | 6 | 6 | 6 | 6 | 40 | 40 | 35 | 27 | 25 | 6 | 6 | 6 | 6 | 40 | 16 |
|  | 46 | 6 | 6 | 6 | 37 | 6 | 40 | 30 | 33 | 26 | 23 | 6 | 6 | 6 | 6 | 6 | 17 |
|  | 59 | 6 | 6 | 6 | 6 | 6 | 40 | 32 | 27 | 27 | 22 | 6 | 6 | 6 | 6 | 40 | 30 |
|  | 62 | 6 | 6 | 6 | 6 | 6 | 38 | 38 | 34 | 26 | 23 | 12 | 6 | 6 | 6 | 40 | 32 |
| Time  period | Isolate Nr. | PEN | CRO | CLI | ERY | SXT | LNZ | TET | TGC | VAN | TEC | MEM | IPM | CIP | GEN | RIF | FA |
| 1994  -1999 | 64 | 6 | 14 | 6 | 6 | 6 | 40 | 28 | 32 | 29 | 28 | 6 | 6 | 6 | 6 | 6 | 12 |
|  | 68 | 6 | 13 | 6 | 6 | 6 | 40 | 33 | 32 | 28 | 24 | 12 | 22 | 6 | 6 | 40 | 18 |
|  | 70 | 6 | 11 | 14 | 40 | 6 | 40 | 38 | 40 | 28 | 23 | 14 | 37 | 6 | 6 | 40 | 22 |
|  | 74 | 6 | 9 | 6 | 6 | 6 | 30 | 19 | 23 | 25 | 22 | 6 | 6 | 6 | 6 | 6 | 15 |
|  | 78 | 6 | 6 | 6 | 6 | 6 | 34 | 36 | 26 | 28 | 24 | 6 | 6 | 6 | 6 | 40 | 14 |
|  | 82 | 6 | 15 | 6 | 15 | 6 | 40 | 40 | 34 | 28 | 24 | 22 | 24 | 6 | 6 | 6 | 30 |
|  | 83 | 6 | 6 | 6 | 6 | 6 | 38 | 28 | 26 | 21 | 21 | 6 | 6 | 6 | 6 | 40 | 14 |
|  | 84 | 6 | 6 | 6 | 6 | 6 | 40 | 26 | 24 | 24 | 22 | 6 | 6 | 6 | 6 | 40 | 12 |
|  | 85 | 6 | 8 | 6 | 6 | 6 | 40 | 34 | 34 | 26 | 22 | 6 | 6 | 6 | 6 | 9 | 16 |
|  | 86 | 6 | 6 | 6 | 6 | 6 | 40 | 29 | 28 | 26 | 23 | 6 | 6 | 6 | 6 | 40 | 14 |
|  | 89 | 6 | 6 | 6 | 14 | 6 | 38 | 37 | 26 | 27 | 24 | 6 | 6 | 6 | 6 | 40 | 15 |
|  | 90 | 6 | 6 | 6 | 14 | 6 | 40 | 28 | 28 | 24 | 22 | 6 | 6 | 6 | 6 | 40 | 14 |
|  | 91 | 6 | 6 | 6 | 6 | 6 | 40 | 28 | 26 | 25 | 22 | 6 | 6 | 6 | 6 | 40 | 16 |
|  | 94 | 6 | 6 | 6 | 8 | 6 | 36 | 33 | 31 | 24 | 22 | 6 | 6 | 6 | 6 | 6 | 40 |
|  | 96 | 6 | 6 | 6 | 6 | 6 | 40 | 36 | 32 | 25 | 22 | 6 | 6 | 6 | 6 | 6 | 16 |
|  | 100 | 6 | 6 | 6 | 13 | 6 | 34 | 34 | 30 | 23 | 21 | 6 | 6 | 6 | 6 | 6 | 15 |
|  | 103 | 6 | 6 | 6 | 6 | 6 | 36 | 26 | 26 | 24 | 22 | 6 | 6 | 6 | 6 | 40 | 14 |
|  | 105 | 6 | 6 | 6 | 6 | 6 | 39 | 38 | 30 | 24 | 21 | 6 | 6 | 6 | 6 | 6 | 25 |
|  | 106 | 6 | 22 | 6 | 6 | 6 | 40 | 26 | 24 | 25 | 22 | 30 | 40 | 6 | 6 | 39 | 14 |
|  | 107 | 6 | 6 | 6 | 6 | 6 | 38 | 34 | 30 | 24 | 22 | 6 | 6 | 6 | 6 | 40 | 30 |
|  | 109 | 6 | 6 | 6 | 6 | 6 | 39 | 28 | 24 | 27 | 24 | 6 | 6 | 6 | 6 | 40 | 16 |
|  | 112 | 6 | 6 | 6 | 6 | 6 | 38 | 34 | 32 | 24 | 22 | 6 | 6 | 6 | 6 | 40 | 30 |
|  | 114 | 6 | 6 | 6 | 6 | 6 | 40 | 34 | 30 | 24 | 22 | 6 | 6 | 6 | 6 | 40 | 30 |
|  | 118 | 6 | 6 | 6 | 17 | 6 | 34 | 27 | 36 | 22 | 20 | 6 | 6 | 6 | 6 | 6 | 26 |
|  | 120 | 6 | 6 | 6 | 6 | 6 | 38 | 36 | 32 | 25 | 21 | 6 | 6 | 6 | 6 | 40 | 13 |
|  | 121 | 6 | 6 | 6 | 6 | 6 | 36 | 36 | 30 | 25 | 21 | 9 | 6 | 6 | 6 | 40 | 30 |
|  | 123 | 6 | 13 | 6 | 6 | 6 | 36 | 39 | 30 | 26 | 22 | 18 | 37 | 6 | 6 | 40 | 15 |
|  | 124 | 6 | 6 | 6 | 6 | 6 | 34 | 33 | 32 | 25 | 21 | 6 | 6 | 6 | 6 | 40 | 28 |
|  | 128 | 6 | 6 | 6 | 6 | 6 | 32 | 33 | 28 | 24 | 21 | 6 | 6 | 6 | 6 | 6 | 11 |
|  | 129 | 6 | 6 | 6 | 6 | 6 | 36 | 40 | 32 | 25 | 40 | 6 | 6 | 6 | 6 | 40 | 40 |
|  | 130 | 6 | 6 | 6 | 12 | 6 | 40 | 18 | 40 | 26 | 22 | 6 | 6 | 6 | 34 | 40 | 15 |
| Time  period | Isolate Nr. | PEN | CRO | CLI | ERY | SXT | LNZ | TET | TGC | VAN | TEC | MEM | IPM | CIP | GEN | RIF | FA |
| 1994  -1999 | 131 | 6 | 7 | 6 | 6 | 6 | 40 | 34 | 31 | 26 | 22 | 6 | 6 | 6 | 6 | 6 | 30 |
|  | 132 | 6 | 6 | 6 | 6 | 6 | 39 | 29 | 24 | 25 | 22 | 6 | 6 | 6 | 6 | 40 | 28 |
|  | 133 | 6 | 11 | 6 | 6 | 6 | 37 | 9 | 30 | 25 | 20 | 17 | 16 | 6 | 18 | 40 | 26 |
|  | 134 | 6 | 6 | 6 | 6 | 6 | 35 | 36 | 30 | 24 | 20 | 6 | 6 | 6 | 6 | 6 | 38 |
|  | 136 | 6 | 6 | 6 | 6 | 6 | 40 | 29 | 24 | 25 | 22 | 6 | 6 | 6 | 6 | 40 | 15 |
|  | 137 | 6 | 6 | 6 | 6 | 6 | 38 | 26 | 24 | 24 | 22 | 6 | 6 | 6 | 6 | 40 | 14 |
|  | 140 | 6 | 6 | 7 | 6 | 6 | 38 | 38 | 32 | 26 | 23 | 6 | 6 | 6 | 6 | 40 | 16 |
|  | 145 | 6 | 6 | 6 | 11 | 6 | 39 | 30 | 28 | 26 | 22 | 6 | 6 | 6 | 6 | 40 | 16 |
| 2012  -2019 | 147 | 6 | 9 | 6 | 6 | 6 | 36 | 40 | 29 | 40 | 23 | 18 | 6 | 28 | 26 | 40 | 14 |
|  | 148 | 6 | 6 | 6 | 6 | 19 | 38 | 37 | 29 | 25 | 22 | 6 | 6 | 6 | 28 | 39 | 12 |
|  | 173 | 6 | 6 | 6 | 6 | 7 | 30 | 32 | 28 | 25 | 22 | 6 | 6 | 6 | 24 | 36 | 12 |
|  | 177 | 6 | 6 | 6 | 6 | 6 | 32 | 31 | 28 | 24 | 24 | 10 | 6 | 6 | 28 | 32 | 30 |
|  | 181 | 6 | 8 | 6 | 6 | 6 | 40 | 36 | 30 | 24 | 20 | 14 | 14 | 6 | 30 | 40 | 28 |
|  | 182 | 6 | 17 | 6 | 9 | 6 | 40 | 32 | 28 | 25 | 24 | 28 | 33 | 6 | 6 | 40 | 40 |

**Supplementary Figure 1: Inhibition zone diameters of penicillin (PEN)**

^a^EUCAST, European Committee on Antimicrobial Susceptibility Testing

^b^CBP, clinical breakpoint for resistance 12 mm; diameters of 12 to 49 mm are susceptible with increased dosage

**Supplementary Figure 2: Inhibition zone diameters of ceftriaxone (CRO)**

^a^ECOFF, epidemiological cutoff; eyeball ECOFF 25 mm

**Supplementary Figure 3: Inhibition zone diameters of clindamycin (CLI)**

^a^EUCAST CBP 20 mm

**Supplementary Figure 4: Inhibition zone diameters of erythromycin (ERY)**

^a^EUCAST CBP 24 mm, taken from *C. diphtheriae* table of EUCAST version 15 (32)

**Supplementary Figure 5: Inhibition zone diameters of trimethoprim-sulfamethoxazole (SXT)**

^a^Eyeball ECOFF 21 mm

**Supplementary Figure 6: Inhibition zone diameters of linezolid (LNZ)**

^a^EUCAST CBP 25 mm

**Supplementary Figure 7: Inhibition zone diameters of tetracycline (TET)**

^a^EUCAST CBP 24 mm

**Supplementary Figure 8: Inhibition zone diameters of tigecycline (TGC)**

^a^Eyeball ECOFF 21 mm

**Supplementary Figure 9: Inhibition zone diameters of vancomycin (VAN)**

^a^EUCAST CBP 17 mm

**Supplementary Figure 10: Inhibition zone diameters of teicoplanin (TEC)**

^a^Eyeball ECOFF 17 mm

**Supplementary Figure 11: Inhibition zone diameters of meropenem (MEM)**

^a^Eyeball ECOFF 21 mm

**Supplementary Figure 12: Inhibition zone diameter of imipenem**

^a^Eyeball ECOFF 25 mm

**Supplementary Figure 13: Inhibition zone diameters of ciprofloxacin (CIP)**

^a^EUCAST CBP for resistance 25 mm; diameters of 25 to 49 mm are susceptible with increased dosage

**Supplementary Figure 14: Inhibition zone diameter of gentamicin (GEN)**

^a^Eyeball ECOFF 23 mm

**Supplementary Figure 15: Inhibition zone diameters of rifampicin (RIF)**

^a^EUCAST CBP 30 mm

**Supplementary Figure 16: Inhibition zone diameters of fusidic acid (FA)**

^a^Eyeball ECOFF 21 mm
